# Longitudinal associations between vitamin D status and biomarkers of inflammation in a pan-European cohort of children and adolescents

**DOI:** 10.1007/s00394-024-03488-7

**Published:** 2024-09-04

**Authors:** Maike Wolters, Ronja Foraita, Luis A. Moreno, Dénes Molnár, Paola Russo, Michael Tornaritis, Stefaan De Henauw, Lauren Lissner, Toomas Veidebaum, Theresa Winter, Manuela Marron, Rajini Nagrani, IDEFICS and I.Family consortia

**Affiliations:** 1 Leibniz Institute for Prevention Research and Epidemiology - BIPS, Achterstrasse 30, 28359 Bremen, Germany; 2 GENUD (Growth, Exercise, Nutrition and Development) Research Group, Instituto Agroalimentario de Aragón (IA2), Instituto de Investigación Sanitaria de Aragón (IIS Aragón), University of Zaragoza, Saragossa, Spain; 3 Centro de Investigación Biomédica en Red de Fisiopatología de la Obesidad y Nutrición (CIBERObn), Instituto de Salud Carlos III, Madrid, Spain; 4 Department of Paediatrics, Medical School, University of Pécs, Pécs, Hungary; 5 Institute of Food Sciences, National Research Council, Avellino, Italy; 6 Research and Education Institute of Child Health, Strovolos, Cyprus; 7 Department of Public Health and Primary Care, Ghent University, Ghent, Belgium; 8 School of Public Health and Community Medicine, Institute of Medicine, Sahlgrenska Academy, University of Gothenburg, Gothenburg, Sweden; 9 National Institute for Health Development, Tallinn, Estonia; 10 Institute of Clinical Chemistry and Laboratory Medicine, University Medicine Greifswald, Greifswald, Germany

**Keywords:** Children cohort, Vitamin D, Inflammatory markers, CRP, Cytokines, Adipokines

## Abstract

**Purpose:**

To investigate longitudinal associations between the vitamin D status and inflammatory markers in children and adolescents.

**Methods:**

Children from eight European countries from the IDEFICS/I.Family cohort with repeated measurements were included in this study. A linear mixed-effect model was used to model the association of serum 25(OH)D as independent variable and z-scores of inflammatory markers [CRP, cytokines, adipokines, combined inflammation score] as dependent variables, where one level accounts for differences between individuals and the other for changes over age within individuals.

**Results:**

A total of 1,582 children were included in the study. In the adjusted model, 25(OH)D levels were positively associated with adiponectin (β = 0.11 [95% CI 0.07; 0.16]) and negatively with the inflammation score (β = − 0.24 [95% CI − 0.40; − 0.08]) indicating that the adiponectin z-score increased by 0.11 units and the inflammation score decreased by 0.24 units per 12.5 nmol/l increase in 25(OH)D. In children with overweight or obesity, only a positive association between 25(OH)D and IP-10 was observed while in children with normal weight adiponectin was positively and the inflammation score was negatively associated. Associations of vitamin D with adiponectin and the inflammation score were stronger in girls than in boys and a positive association with TNF-α was observed only in girls.

**Conclusion:**

Our results suggest that an increase in vitamin D concentrations may help to regulate inflammatory biomarkers. However, it seems to be no benefit of a better vitamin D status in children with overweight/obesity unless their weight is managed to achieve an improved inflammatory marker status.

**Supplementary Information:**

The online version contains supplementary material available at 10.1007/s00394-024-03488-7.

## Introduction

Obesity is widespread, in children and adolescents, and increases the risk of metabolic disturbances at young age [1–3]. Children with abdominal obesity are at high risk of developing metabolic syndrome outcomes such as hypertension, dyslipidemia and dysglycemia which mostly persist into adolescence [2, 4] and adulthood [5, 6]. Adipose tissue has been shown to be an immunologically active organ which—in an obese status—secretes a large quantity of pro-inflammatory cytokines and adipokines such as tumor necrosis factor alpha (TNF-α), interleukin 6 (IL-6), interferon gamma inducible protein (IP-10) and leptin. This leads to chronic systemic inflammation, additionally mediated by macrophages and B lymphocytes, which reduces insulin sensitivity and contributes to the increased risk of several metabolic disorders [7, 8].

Vitamin D exerts immunomodulatory properties and can inhibit chronic systemic inflammation. It is involved in the function of immunocompetent cells such as macrophages, monocytes, dendritic cells as well as B and T lymphocytes [9]. A systematic review of *in-vitro* studies investigating the effect of vitamin D on the protein expression and secretion of inflammatory markers by human-derived immune cell lines or peripheral blood mononuclear cells found mainly anti-inflammatory effects, like the suppression of IL-6 and IL-8 indicating that different mechanisms may be responsible for these anti-inflammatory properties. For instance, vitamin D decreased the expression of toll-like receptor (TLR)-2 and TLR-4 and decreased the concentrations of reactive oxygen species [10]. In line with these results, leptin and IL-6 expression as well as nuclear factor-κB and extracellular signal-regulated kinase-1/2 (ERK1/2) phosphorylation were suppressed in human adipocytes by vitamin D through its receptor (VDR) [11]. Accordingly, serum 25-hydroxyvitamin D [25(OH)D] as the established biomarker of the vitamin D status [12] has been shown to be inversely associated with pro-inflammatory cytokines such as high sensitive serum C-reactive protein (CRP) [13] and IL-6 [14]. However, results are contradictory and the association seems to depend on 25(OH)D concentrations and adiposity. In 358 healthy men, CRP showed a strong inverse correlation with 25(OH)D only up to 14 ng/ml [15]. In a large cohort of asymptomatic adults, an inverse relation between CRP and 25(OH)D at concentrations < 21 ng/ml was found. At a concentration ≥ 21 ng/ml, 25(OH)D was associated with an increase in serum CRP [16]. Hypponen et al. [17] found that CRP and 25(OH)D concentrations adjusted for sex and month of blood draw were inversely associated in adults, but this association was explained by adiposity. A meta-analysis of randomized controlled trials (RCTs) did not show an inverse effect of vitamin D supplementation on IL-6 or CRP. However, subgroup analysis of trials achieving a serum 25(OH)D concentration of ≥ 80 nmol/l and trials with vitamin D dosages of ≥ 1000 IU/d indicated a potentially lowering effect on CRP although there was substantial heterogeneity [18].

Studies on the relation of the vitamin D status and inflammatory markers in children and adolescents are scarce. In particular, longitudinal studies and studies covering numerous different inflammatory markers in this population are missing. Several cross-sectional studies indicated associations between serum 25(OH)D concentration and inflammatory markers: In Spanish school children, low serum 25(OH)D was associated with high IL-6 in children with overweight/obesity as well as with increased CRP in children with obesity [19]. In another Spanish study in children with obesity and insufficient vitamin D status, elevated IL-6 concentrations were observed [20]. In contrast, the 25(OH)D concentration was positively associated with CRP in 263 Danish school children [21] whereas no association with CRP, IL-6 and TNF-α was reported in school children from Ireland [22]. Also in a systematic review aiming to evaluate the association of vitamin D status and inflammation in children and adolescents, contradictory results were observed as three of six studies indicated an inverse association of the vitamin D status with biomarkers of inflammation, i.e. CRP, IL-6 or adiponectin, whereas the other three showed no association [23]. In a longitudinal study in two mother–child cohorts, positive associations between 25(OH)D at age 6 months and hs-CRP at 4, 6, and 12 years were detected, whereas cross-sectionally a negative association was reported at age 6 months [24].

Concentrations of pro-inflammatory markers are increased [25, 26] and vitamin D concentration is decreased [27, 28] with excess body fat, BMI and waist circumference. Therefore, overweight/ obesity and vitamin D deficiency need to be considered when analysing the association. Thus, this study aims to answer the following research questions in a large cohort of European children and adolescents: (i) Are there longitudinal associations of serum 25(OH)D concentrations with single inflammatory markers (adiponectin, leptin, ghrelin, CRP, IL-1Ra, IL-6, IL-8, IL-15, IP-10, TNF-α and a derived inflammation score) adjusting for weight status and other relevant covariables?; (ii) Do the associations differ between children with normal weight and children with overweight/obesity?

## Methods

### Study population

Children and adolescents of the IDEFICS/I.Family cohort from eight European countries (Belgium, Cyprus, Estonia, Germany, Hungary, Italy, Spain and Sweden) were included in this study. In the baseline survey (Wave 1 = T_0_, 2007/2008), 16,229 children aged 2–9 years participated. Two years later (Wave 2 = T_1_, 2010/2011) and six years later (Wave 3 = T_3_, 2013/2014), follow-up examinations were conducted. The present study includes data from a subsample of T_0_ and T_3_. The IDEFICS/I.Family study investigated health-related behaviours, biomarkers and health outcomes in children and adolescents. The children underwent thorough physical examinations and collection of blood samples after an overnight fast of at least 8 h at all survey waves. Additionally, questionnaires on health-related behaviours including physical activity, media use and dietary intake were completed by a parent for children aged up to 11 years while children aged 12 years and older completed the questionnaires for themselves. All assessments and procedures were standardized following standard operating procedures and were applied consistently at each survey wave. Written informed consent was obtained from parents and children aged 12 years and older before the start of the study while younger children gave oral consent. Prior to the start of the study, ethics approval from the institutional review boards was obtained in all eight study centres. More detailed information on the study design can be found elsewhere [29, 30].

### 25-Hydroxyvitamin D

For the measurement of serum 25(OH)D concentrations, the chemiluminescence assay, IDS-iSYS 25-Hydroxy Vitamin Dˢ (Immunodiagnostic Systems Ltd, Boldon, United Kingdom), was used and all samples were measured in the central laboratory of the University medicine Greifswald (UMG) at the Institute of Clinical Chemistry and Laboratory Medicine. Prior to the measurements, the samples were stored at − 80 °C in the Integrated Research Biobank of the UMG [31]. The measurement range is 10 to 275 nmol/l (4–110 ng/ml) and is aligned to the National Institute of Standards and Technology Standard Reference Material, (NIST SRM) 2972 (NIST, Gaithersburg, Maryland, USA) according to the manufacturer information.

### Inflammatory markers

The concentrations of inflammatory markers were measured in serum samples drawn at T_0_ and T_3_ which were stored at − 80° until analysis. ELISA using electrochemiluminescent multiplex assay (using either single or MULTI-SPOT® Assay Systems, Meso Scale Discovery, Rockville, USA) was used to detect concentrations of inflammatory markers. While IL-1Ra, IL-6, IL-8, IL-15, TNF-α and IP-10 were run together on a 6-plex cytokine assay kit, leptin ran together with insulin on a 2-plex assay. Adiponectin, ghrelin and CRP were run on single-plex assays each.

### Anthropometric measures

Body weight and height were measured with light clothes and no shoes. Weight was measured to the nearest 0.1 kg using a TANITA digital scale (TANITA Europe GmbH, Sindelfingen, Germany), height to the nearest 0.1 cm using a stadiometer (Seca GmbH & Co. KG., Hamburg, Germany). In the analyses, age and sex-specific BMI z-scores were used based on Cole and Lobstein [32]. Measurement of waist circumference was conducted midway between the lowest rib margin and the iliac crest to the nearest 0.1 cm in upright position with relaxed abdomen and feet together using a non-elastic tape (Seca 200; seca, Birmingham, UK).

### Pubertal status and health-related behaviours

The pubertal status of children (pre-pubertal vs. pubertal) was defined based on age at menarche in girls and voice change in boys based on a self-reported questionnaire completed by children from the age of 8 years in T_3_. This definition has been shown to provide similar results when compared to Tanner stages according to a previous analysis in this cohort [33].

A healthy diet adherence score (HDAS) was used to assess diet quality based on a validated and reproducible food frequency questionnaire (FFQ) as previously described [34]. The composite score is derived from the consumption frequency of fruits and vegetables, refined sugars, fat, whole meal products and fish. A higher score reflects a higher adherence to dietary guidelines which are common across all participating countries [35]. For the present analysis the score was used as a continuous variable. Smoking habits and alcohol consumption were self-reported by children aged 12 years and older at T_3_. Based on the number of occasions reported for alcohol intake/cigarette smoking in lifetime, binary indicator variables were created for alcohol intake and smoking of ever smokers/drinkers vs. non-smokers/non-drinkers. As a proxy for physical activity, a variable indicating whether the child was member in a sports club (yes/no) was used. Total screen time including TV, DVD, video, computer or games-console use in hours for the whole week was used as a proxy for sedentary behaviour.

### Demographic factors, parental education and early life factors

In all analyses, age and sex of the children as well as country of residence were considered as covariates. The highest educational level of parents according to the International Standard Classification of Education (ISCED) was used an indicator for socioeconomic status and was grouped into three categories: low (ISCED 0, 1, 2); medium (ISCED 3, 4) and high level (ISCED 5, 6) [36]. Supplementary Table 1 describes the assessment of early life factors including maternal weight status, preterm birth, birth weight and breastfeeding duration.

### Analysis dataset

Measurement of 25(OH)D and of at least one inflammatory marker at T_0_ and/or T_3_ was available for 1997 participants. Intake of anti-inflammatory medication or corticosteroids (n = 188) was an exclusion criterion. Additionally, we excluded children with serum CRP ≥ 10 mg/l (n = 227) to rule out participants with acute bacterial or viral infection, asthma, autoimmune inflammatory disorders such as rheumatoid arthritis or other unrecognized systemic diseases. Thus, our analysis dataset included 1582 study participants (Supplementary Fig. 1).

### Statistical analysis

For continuous variables, data are shown as median with 25th and 75th percentiles whereas categorical variables are expressed as frequencies (percentages). The stata module STNDZXAGE [37] was used for calculating z-scores of inflammatory markers by standardizing its raw values (irrespective of their distribution with respect to the detection limits) over age, sex, and survey. To assess the combined effect on all inflammatory markers, an inflammation score was calculated by subtracting the sum of z-scores of the anti-inflammatory markers (IL-1Ra, adiponectin, ghrelin, IL-15) from the sum of z-scores of the pro-inflammatory markers (leptin, CRP, TNF-α, IP-10, IL-8, IL-6) such that the lower inflammation score depicted healthy inflammation profile. The longitudinal association between 25(OH)D and inflammatory markers was modelled, using a linear mixed-effect model, where one level accounts for differences between individuals and the other level for changes over age within individuals [38]. The exposure, serum concentration of 25(OH)D, and the outcomes, the inflammatory markers, were used as continuous variables. The between-subject effect estimate represented the longitudinal association between 25(OH)D and the single inflammatory marker, the fixed-effect interaction between age and 25(OH)D referred to the rate of change in the association between 25(OH)D and inflammatory markers per year increase in age.

Crude models included age and sex as potential confounding variables. In the adjusted models, the following variables were additionally included: study region as a proxy for ethnicity, lifetime smoking and alcohol status, membership in a sports club (proxy for physical activity), and screen time per week (proxy for sedentary behaviour), BMI, month of blood sample collection and parental education status. To evaluate the marginal effect of 25(OH)D on inflammatory markers at different ages we conducted post-hoc analyses using effect estimates from the adjusted model. We evaluated the modifying effects of categorical variables of sex and BMI [32] through (i) stratified analysis and (ii) assessing multiplicative interactions with vitamin D.

Several sensitivity analyses were conducted: (i) 25(OH)D concentrations were categorized into deficient (< 50 nmol/l), insufficient (50–< 75 nmol/l) and normal (75–125 nmol/l) to compare our findings with the previously used cut-offs for vitamin D status [39, 40]. (ii) Non-linear relationship between 25(OH)D and inflammatory markers was evaluated using linear spline with knots at 25, 50, 75 and 100 nmol/l. (iii) Further, we additionally adjusted for metabolic and early life factors and the diet quality on our adjusted models to evaluate if factors such as pubertal status, birth weight, duration of breastfeeding, preterm birth, maternal obesity and diet quality attenuate the association.

All covariates were treated as time-varying in order to account for changes in health-related behaviours and BMI over time. Results were reported as regression coefficients with 95% confidence intervals. We accounted for multiple testing in the main analysis using Bonferroni correction, that is the statistical significance level was set to α = 0.05/11 = 0.005 (10 inflammatory markers and 1 inflammation score). All statistical analyses were performed using Stata 17.

## Results

### Characteristics of the study population

Table 1 shows the characteristics of the study population. In total, 1582 children were included in the study. Their mean age was 6.6 years at baseline and 12.4 years at follow-up examination. The mean serum concentrations of 25(OH)D were 44.3 nmol/l at baseline and 44.5 nmol/l at follow-up examination. Only 1.9% of children at baseline and 3.7% at follow-up examination had vitamin D levels within the normal range. The percentage of children with overweight or obesity was 15.1% and 21.5% at baseline and follow-up examinations, respectively. This increase is also reflected by the higher mean BMI z-score of 0.22 at follow-up versus − 0.09 at baseline. Serum CRP concentrations were higher at baseline compared to follow-up examination. This was also true for the anti-inflammatory markers IL-1Ra and ghrelin. In contrast, leptin and IL-8 concentrations were considerably higher at follow-up than at baseline examination. More children were member in a sports club at follow-up examination and the screen time per week was increased at follow-up compared to baseline examination. Table 2 shows the 25(OH)D concentrations and the deficient/insufficient versus normal status by age group and weight group. Supplementary Table 2 shows the serum concentrations of the inflammatory markers by weight status at baseline and follow-up.

**Table 1:** Characteristics of the analysis group at baseline and follow-up examinations

Parameters	IDEFICS/I.Family cohort (n = 1582)	
	Baseline; T_0_ (n = 1566)	Follow-up examination; T_3_ (n = 1581)
Sex, female: n (%)	745 (47.6)	749 (47.4)
Age: years	6.6 (4.7; 7.8)	12.4 (10.5; 13.6)
Study region		
Italy: n (%)	211 (13.5)	213 (13.5)
Estonia: n (%)	300 (19.2)	305 (19.3)
Cyprus: n (%)	34 (2.2)	35 (2.2)
Belgium: n (%)	83 (5.3)	83 (5.3)
Sweden: n (%)	196 (12.5)	195 (12.3)
Germany: n (%)	268 (17.1)	270 (17.1)
Hungary: n (%)	397 (25.4)	398 (25.2)
Spain: n (%)	77 (4.9)	82 (5.2)
BMI^a^		
Thinness grade 1–3: n (%)	187 (11.9)	133 (8.4)
Normal weight: n (%)	1143 (73.0)	1108 (70.1)
Overweight/obese: n (%)	236 (15.1)	340 (21.5)
25-Hydroxyvitamin D		
Normal 75–125 nmol/l: n (%)	30 (1.9)	58 (3.7)
Insufficient 50– < 75 nmol/l: n (%)	382 (24.4)	481 (30.4)
Deficient < 50 nmol/l: n (%)	745 (47.6)	945 (59.8)
High > 125 nmol/l	0 (0.0)	7 (0.4)
missing	409 (26.1)	90 (5.7)
Pubertal status: n (%)	0 (0)	693 (43.83)
Ever smoking^b^: n (%)	Not observed	108 (6.83)
Ever consumed alcohol^b^: n (%)	Not observed	305 (19.29)
Membership in sports club: n (%)	779 (49.74)	1048 (66.29)
HDAS, n(T_0_) = 1527, n(T_3_) = 1488	21 (15; 26)	18 (13; 23)
Screen time per week (hours), n(T_0_) = 1510, n(T_3_) = 1476	10.5 (6.8; 15.3)	15.3 (9.3; 22.8)
25-Hydroxyvitamin D (nmol/l), n(T_0_) = 1157, n(T_3_) = 1491	44.3 (33.5; 54.3)	44.5 (33.8; 55.0)
BMI z-score	− 0.09 (− 0.84; 0.86)	0.22 (− 0.64; 1.57)
Adiponectin (µg/ml), n(T_0_) = 531, n(T_3_) = 1224	24.64 (18.77; 33.45)	22.12 (15.68; 33.63)
Leptin (ng/ml), n(T_0_) = 820, n(T_3_) = 1547	1.48 (0.90; 2.72)	4.67 (2.07; 1.17)
Ghrelin (pg/ml), n(T_0_) = 641, n(T_3_) = 1130	55.00 (26.74; 93.78)	38.57 (16.7; 68.5)
CRP (mg/l), n(T_0_) = 868, n(T_3_) = 1528	1.01 (0.33; 2.51)	0.25 (0.08; 0.81)
IL-1Ra (pg/ml), n(T_0_) = 833, n(T_3_) = 1460	314.38 (206.26; 462.36)	264.8 (196.74; 368.87)
IL-6 (pg/ml), n(T_0_) = 815, n(T_3_) = 1449	0.28 (0.17; 0.47)	0.40 (0.28; 0.58)
IL-8 (pg/ml), n(T_0_) = 834, n(T_3_) = 1466	3.21 (2.28; 4.58)	6.32 (4.67; 9.06)
IL-15 (pg/ml), n(T_0_) = 827, n(T_3_) = 1466	1.86 (1.25; 2.63)	2.28 (1.77; 2.89)
IP-10 (ng/ml), n(T_0_) = 834, n(T_3_) = 1467	0.18 (0.13; 0.26)	0.21 (0.16; 0.30)
TNF-α (pg/ml), n(T_0_) = 834, n(T_3_) = 1466	2.20 (1.70; 2.93)	2.45 (1.98; 3.15)
Adiponectin z-scores, n(T_0_) = 531, n(T_3_) = 1224	− 0.17 (− 0.35; 0.10)	− 0.31 (− 0.54; 0.11)
Leptin z-scores, n(T_0_) = 820, n(T_3_) = 1547	− 0.32 (− 0.55; 0.17)	− 0.33 (− 0.48; 0.08)
Ghrelin z-scores, n(T_0_) = 641, n(T_3_) = 1130	− 0.11 (− 0.17; 0.00)	− 0.13 (− 0.19; − 0.02)
CRP z-scores, n(T_0_) = 868, n(T_3_) = 1528	− 0.38 (− 0.68; 0.29)	− 0.40 (− 0.52; 0.03)
IL-1Ra z-scores, n(T_0_) = 833, n(T_3_) = 1460	− 0.23 (− 0.56; 0.31)	− 0.26 (− 0.58; 0.23)
IL-6 z-scores, n(T_0_) = 815, n(T_3_) = 1449	− 0.11 (− 0.22; 0.02)	− 0.15 (− 0.28; 0.02)
IL-8 z-scores, n(T_0_) = 834, n(T_3_) = 1466	− 0.07 (− 0.18; 0.05)	− 0.10 (− 0.14;− 0.02)
IL-15 z-scores, n(T_0_) = 827, n(T_3_) = 1466	− 0.17 (− 0.67; 0.50)	− 0.16 (− 0.65; 0.45)
IP-10 z-scores, n(T_0_) = 834, n(T_3_) = 1467	− 0.25 (− 0.39; − 0.01)	− 0.21 (− 0.55; 0.30)
TNF-α z-scores, n(T_0_) = 834, n(T_3_) = 1466	− 0.17 (− 0.68; 0.48)	− 0.20 (− 0.34; 0.00)
Inflammation score z-score, n(T_0_) = 330, n(T_3_) = 842	− 0.28 (− 1.31; 0.84)	− 0.33 (− 1.51; 0.90)

BMI, body mass index; CRP, C-reactive protein; HDAS, healthy diet adherence score; IL, interleukin; IL-1Ra, interleukin-1 receptor antagonist; IP-10, interferon gamma inducible protein; TNF-α, tumor necrosis factor alpha

^a^Category for BMI was calculated using Cole and Lobstein, 2012

^b^Children below 12 were assumed to be non-smokers and non-drinkers. Characteristics of the study participants are presented as number (percentages) for categorical variables and median (25th and 75th percentiles) for continuous variables. n stated in case of missingness

**Table 2:** Serum 25-hydroxyvitamin D concentration and vitamin D status by age group and weight status

Examination	25-Hydroxyvitamin D (nmol/l)^a^		Deficient/insufficient 25(OH)D (< 75 nmol/l), n (%)		Normal 25(OH)D (75–125 nmol/l), n (%)	
	Baseline; T_0_ (n = 1566)	Follow-up; T_3_ (n = 1581)	Baseline; T_0_ (n = 1127)	Follow-up; T_3_ (n = 1426)	Baseline; T_0_ (n = 30)	Follow-up; T_3_ (n = 58)
Age group						
2 to < 6 years (pre-school)	45.0 (34.5; 55.5)	na	459 (40.7)	na	14 (46.7)	na
6 to < 10 years (primary school)	43.5 (32.3; 53.3)	50.8 (40; 61.3)	668 (59.3)	215 (15.1)	16 (53.3)	16 (27.6)
10 to ≤ 16 years (teens)	na	43.8 (33.0; 54.0)	na	1211 (84.9)	na	42 (72.4)
Weight status						
Underweight	43.6 (33.9; 54.4)	47.0 (36.3; 56.5)	148 (13.1)	122 (8.6)	0 (0.0)	2 (3.5)
Normal weight	44.0 (33.5; 54.3)	45.0 (34.3; 55.5)	839 (74.5)	999 (70.1)	24 (80.0)	43 (74.1)
Overweight/obese	45.8 (32.8; 54.8)	42.0 (32.5; 52.8)	140 (12.4)	305 (21.4)	6 (20.0)	13 (22.4)
Missing; n(%)	409 (26.1)	90 (5.7)	–	–	–	–

25(OH)D, 25-hydroxyvitamin D; na, not applicable

^a^Median (25th and 75th percentiles). Note: Seven observations from follow-up examination (T_3_) were excluded from this category of vitamin D status as 25(OH)D levels > 125nmol/l

### Associations between serum 25(OH)D and inflammatory markers

**Fig. 1:**
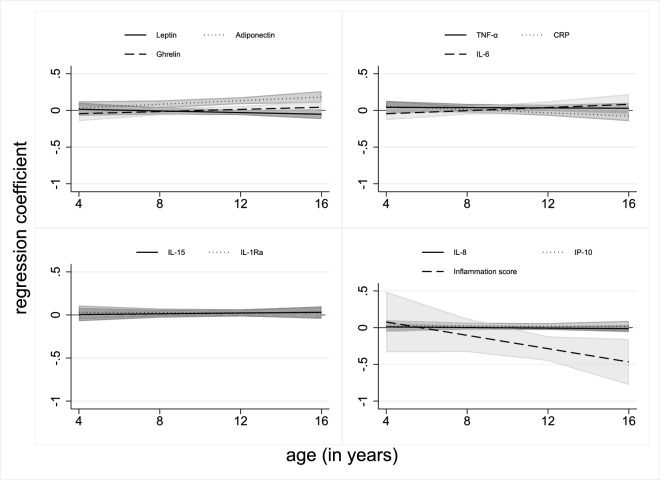
Posthoc analyses to evaluate the marginal effect of 25(OH)D on inflammatory markers at different ages. 25(OH)D, 25-hydroxyvitamin D; CI, confidence interval; CRP, C-reactive protein; IL, interleukin; IL-1Ra, interleukin-1 receptor antagonist; IP-10, interferon gamma inducible protein; TNFα, tumor necrosis factor alpha. Inflammation score = sum of z-scores of proinflammatory markers (CRP, leptin, TNF-α, IP-10, IL-8, IL-6) - sum of z-scores of anti-inflammatory markers (IL-1Ra, IL-15, adiponectin, ghrelin). Marginal effects are presented as regression coefficient that represent the *ß* unit change in the z-score of inflammatory markers per 12.5 nmol/l increase in 25(OH)D at different ages. Associations are adjusted for age, sex, study region, lifetime smoking and alcohol status, membership in sports club, screen time/week, BMI, month of blood sample collection and parental education status with interaction term between 25(OH)D and age with age as a random slope

Table 3 depicts the associations between serum 25(OH)D and inflammatory markers. In the crude model, serum 25(OH)D concentrations were positively associated with adiponectin, IL-8 and TNF-α but inversely with leptin and the inflammation score. In the adjusted model, associations remained for adiponectin (β = 0.11 [95% CI 0.07; 0.16]) and the inflammation score (β = − 0.24 [95% CI − 0.40; − 0.08]) indicating that the adiponectin z-score increased by 0.11 units and the inflammation score decreased by 0.24 units per 12.5 nmol/l increase in 25(OH)D. Additionally, an interaction with age was observed for adiponectin and CRP indicating that the associations strengthened with increasing age. These interactions are also reflected in Fig. 1 which displays the association of 25(OH)D and inflammatory markers at different ages. Further, additional adjustment for early life markers and for the quality of the diet did not change the association of 25(OH)D with adiponectin and slightly strengthened the association with the inflammation score (Supplementary Table 3). In Supplementary Fig. 2 the non-linear associations between 25(OH)D concentrations and inflammatory markers are shown. There were positive associations with adiponectin when 25(OH)D concentrations were ≥ 100 nmol/l and a weak positive association with IP-10 when 25(OH)D concentrations were 50–< 75 nmol/l.

**Table 3:** Associations between serum 25-hydroxyvitamin D and inflammatory markers

Inflammatory markers	Crude^a^		Adjusted^b^		Interaction with age^c^
	*n*	*β* (95% CI)	*N*	*β* (95% CI)	*β* (95% CI)
Adiponectin	1266	**0.13 (0.09; 0.16)**	1150	**0.11 (0.07; 0.16)**	**0.01 (0.0002; 0.02)**
Leptin	1541	**− 0.06 (− 0.09; –0.02)**	1430	− 0.02 (− 0.06; 0.01)	− 0.01 (− 0.02; 0.01)
Ghrelin	1221	− 0.01 (− 0.04; 0.02)	1119	0.00 (− 0.03; 0.04)	0.01 (− 0.01; 0.02)
CRP	1530	− 0.02 (− 0.05; 0.01)	1426	− 0.01 (− 0.05; 0.02)	**− 0.01 (− 0.02; − 0.001)**
IL-1Ra	1503	0.00 (− 0.03; 0.03)	1393	0.03 (− 0.01; 0.06)	− 0.001 (− 0.01; 0.01)
IL-6	1488	− 0.01 (− 0.05; 0.03)	1380	0.00 (− 0.06; 0.05)	0.01 (− 0.01; 0.03)
IL-8	1505	**0.02 (0.00; 0.04)**	1396	0.00 (− 0.02; 0.02)	− 0.002 (− 0.01; 0.01)
IL-15	1505	0.02 (− 0.01; 0.05)	1396	0.02 (− 0.02; 0.06)	0.002 (− 0.01; 0.01)
IP-10	1505	− 0.02 (− 0.05; 0.01)	1396	0.02 (− 0.02; 0.06)	− 0.0003 (− 0.01; 0.01)
TNF-α	1505	**0.05 (0.01; 0.08)**	1396	**0.04 (0.00; 0.08)**	− 0.001 (− 0.01; 0.01)
Inflammation score	903	**− 0.28 (− 0.42; − 0.15)**	810	**− 0.24 (− 0.40; − 0.08)**	− 0.05 (− 0.10; 0.01)

Inflammation score = sum of *z*‐scores of proinflammatory markers (CRP, leptin, TNF-α, IP‐10, IL‐8, IL‐6) − sum of *z*‐scores of anti‐inflammatory markers (IL‐1Ra, IL‐15, adiponectin, ghrelin). The *ß* coefficient represents the *ß* unit change in the z-score of inflammatory markers per 12.5 nmol/l increase in 25(OH)D. Associations at *p* < 0.05 are shown in bold. Associations after Bonferroni correction (*p* < 0.005) are shown in bold and italics

25(OH)D, 25-hydroxyvitamin D; CI, confidence interval; CRP, C-reactive protein; IL, interleukin; IL-1Ra, interleukin-1 receptor antagonist; IP-10, interferon gamma inducible protein; TNFα, tumor necrosis factor alpha

^a^Crude model is adjusted for age and sex with age as a random slope

^b^Adjusted model is additionally adjusted for study region, lifetime smoking and alcohol status, membership in sports club, screen time/week, BMI, month of blood sample collection and parental education status

^c^p-value of adjusted model for interaction with age

### The role of weight status, vitamin D insufficiency or deficiency and sex

**Fig. 2:**
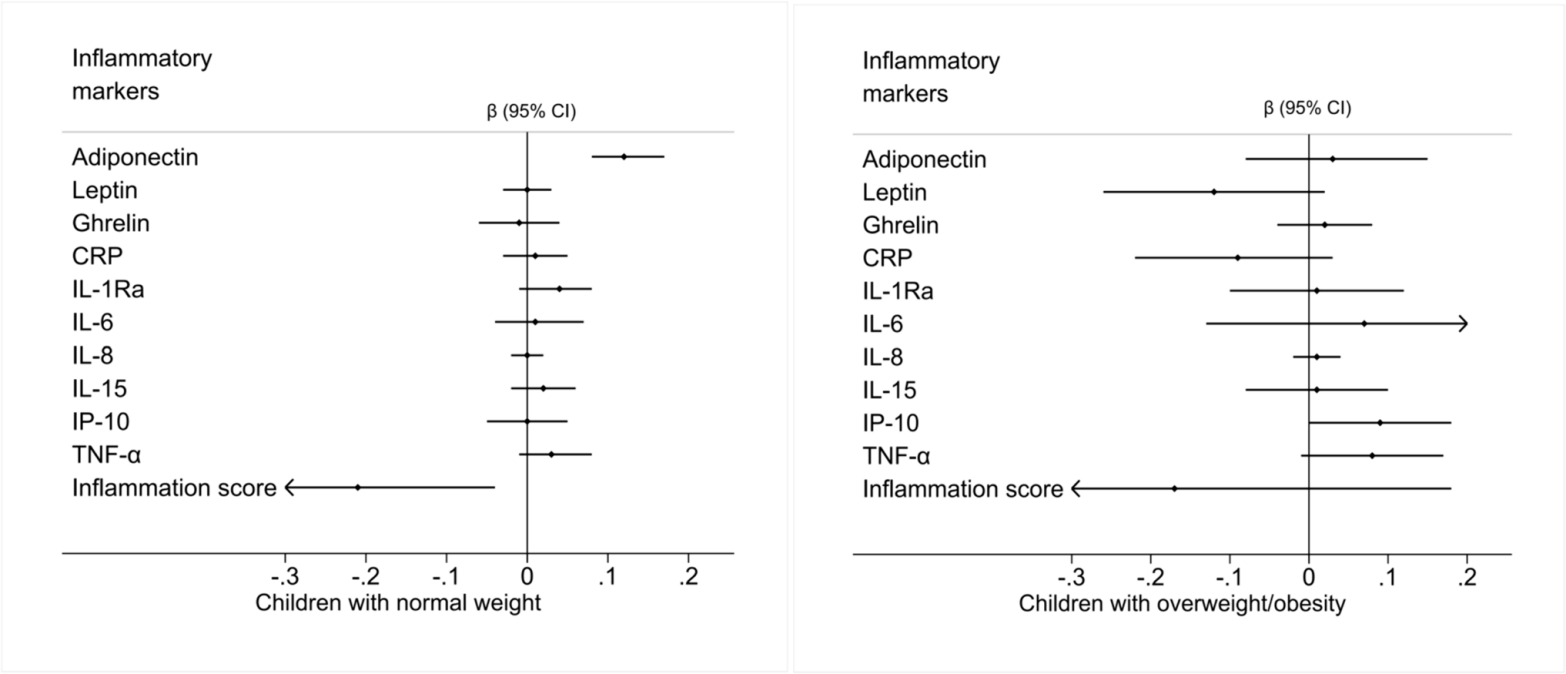
Association between serum 25-hydroxyvitamin D and inflammatory markers stratified on BMI. Abbreviations: 25(OH)D, 25-hydroxyvitamin D; BMI, body mass index; CI, confidence interval; CRP, C-reactive protein; IL, interleukin; IL-1Ra, interleukin-1 receptor antagonist; IP-10, interferon gamma inducible protein; TNF-α, tumor necrosis factor alpha. Inflammation score = sum of *z-*scores of proinflammatory markers (CRP, leptin, TNF-α, IP-10, IL-8, IL-6) − sum of *z*-scores of anti-inflammatory markers (IL-1Ra, IL-15, adiponectin, ghrelin). Normal weight children: 18.5 ≤ BMI < 25; Overweight/obese children: BMI ≥ 25 [32]. The *ß* coefficient represents the *ß* unit change in the z-score of inflammatory markers per 12.5 nmol/l increase in 25(OH)D. Associations are adjusted for age, sex, study region, lifetime smoking and alcohol status, membership in sport club, screen time/week, month of blood sample collection and parental education status with age as a random slope

Figure 2 and Supplementary Table 4 present the associations between 25(OH)D concentrations and inflammatory markers by weight status. In normal weight children, an increase in 25(OH)D concentrations was positively associated with adiponectin and inversely associated with the inflammation score similar to the results in the whole study population. In children with overweight or obesity, a positive association between 25(OH)D and IP-10 was observed. In Table 4, the associations of 25(OH)D and inflammatory markers in children with insufficient and deficient 25(OH)D compared to normal status are shown. Supplementary Table 5 presents the associations between 25(OH)D and inflammatory markers stratified by sex. The positive association with adiponectin was stronger in girls than in boys. Additionally, in girls, 25(OH)D was positively associated with TNF-α. There was a strong inverse association with the inflammation score in girls (β = − 0.44 [95% CI − 0.66; − 0.22]) which accordingly showed an interaction with sex.

**Table 4:** Association between 25-hydroxyvitamin D and inflammatory markers in children with insufficient and deficient 25(OH)D compared to children with normal 25(OH)D levels

Inflammatory markers	*n*	Insufficient^a^	Deficient^a^
		*β* (95% CI)	*β* (95% CI)
Adiponectin	1145	0.12 (− 0.20; 0.44)	− 0.03 (− 0.35; 0.29)
Leptin	1423	0.18 (− 0.05; 0.42)	0.20 (− 0.03; 0.44)
Ghrelin	1116	0.07 (− 0.17; 0.32)	0.05 (− 0.20; 0.30)
CRP	1419	− 0.06 (− 0.31; 0.19)	0.01 (− 0.24; 0.27)
IL-1Ra	1386	0.10 (− 0.17; 0.36)	− 0.03 (− 0.30; 0.23)
IL-6	1373	0.09 (− 0.28; 0.46)	0.05 (− 0.33; 0.42)
IL-8	1389	− 0.06 (− 0.22; 0.10)	− 0.02 (− 0.18; 0.14)
IL-15	1389	0.05 (− 0.21; 0.32)	− 0.04 (− 0.30; 0.22)
IP-10	1389	− 0.05 (− 0.32; 0.22)	− 0.16 (− 0.43; 0.11)
TNF-α	1389	− 0.11 (− 0.40; 0.17)	− 0.19 (− 0.48; 0.09)
Inflammation score	808	0.40 (− 0.76; 1.55)	0.89 (− 0.25; 2.03)

Inflammation score = sum of *z*-scores of proinflammatory markers (CRP, leptin, TNF-α, IP-10, IL-8, IL-6) − sum of *z*-scores of anti-inflammatory markers (IL-1Ra, IL-15, adiponectin, ghrelin). The *ß* coefficient represents the *ß* unit change in the z-score of inflammatory markers in children with insufficient and deficient 25(OH)D compared to normal 25(OH)D levels. Associations at *p* < 0.05 are shown in bold. Associations are adjusted for age, sex, study region, lifetime smoking and alcohol status, membership in sport club, screen time/week, BMI, month of blood sample collection and parental education status with age as a random slope

25(OH)D, 25-hydroxyvitamin D; CI, confidence interval; CRP, C-reactive protein; IL, interleukin; IL-1Ra, interleukin-1 receptor antagonist; IP-10, interferon gamma inducible protein; TNFα, tumor necrosis factor alpha

^a^deficient (< 50 nmol/l), insufficient (50–< 75 nmol/l), normal (75–125 nmol/l). Note: 7 observations from follow-up examination (T_3_) were excluded from this analysis due to 25(OH)D levels > 125nmol/l

## Discussion

This study evaluated the longitudinal relationship of serum 25(OH)D concentrations and a set of inflammatory markers in a pan-European cohort of over 1,500 children and adolescents. Our results demonstrate a positive association between 25(OH)D and adiponectin concentrations, and an inverse association between 25(OH)D and inflammation score, confirming anti-inflammatory role of vitamin D. These associations were stronger in girls and limited to children with normal weight.

Contrary to our findings of 25(OH)D with adiponectin, a cross-sectional study of over 800 Danish children aged 8–11 years found no association between 25(OH)D and adiponectin [41]. Similarly, two previous systematic reviews and meta-analyses of RCTs did not find an effect of vitamin D supplementation on adiponectin concentrations [42, 43]. However, in one of the meta-analysis a small increase was reported in adiponectin levels if supplementation was given on a daily basis and in patients with diabetes [42]. Except for adiponectin, we did not observe associations with other single pro- or anti-inflammatory markers. This aligns with studies from Denmark and the US [41, 44], but contrasts with findings from Spain [19], China [45] and another US study [46], which reported association of 25(OH)D with IL-6 and CRP. Similar to our results, no associations with CRP, IL-6, IL-8 and TNF-α were detected in Irish school children [22], while in another small sample of children with obesity who had an insufficient vitamin D status, IL-6 was substantially elevated [20]. A systematic review and meta-analysis of RCTs investigating the effect of vitamin D supplementation on systemic inflammatory markers indicated that a high dosage of vitamin D (≥ 1000 IU/d) seems to be required to achieve a reduction of CRP [18]. Accordingly, a pilot study in adults suggests that high vitamin D concentrations above 100 nmol/L following vitamin D supplementation may reversibly reduce TLR2 stimulated cytokines for TNF-α, IL-6 and IFN-α which indicates that vitamin D may down-regulate the innate immune system [47].

Our stratified analysis and interaction analysis with BMI indicated that increased weight status attenuated the associations between 25(OH)D levels and adiponectin. This is not surprising as obesity is a strong confounder in the association between vitamin D and adipokines and cytokines [48]. As observed in the present and previous studies, serum 25(OH)D [27, 28] and adiponectin concentrations [49, 50] are lower in children with obesity compared to children with normal weight, further, the strong inverse association between BMI and adiponectin [51] may have weakened the association in children with overweight/ obesity in our study. However the literature is inconsistent on association between vitamin D levels and adiponectin, for example, a small German study reported a positive association between 25(OH)D and adiponectin concentrations in children with obesity [52], whereas, two RCTs in adolescents with overweight and/or obesity did not find significant effects of vitamin D supplementation on adiponectin [53, 54]. Further, proteomics data from a study in children with obesity with either deficient or normal vitamin D status indicated that adiponectin is reduced in vitamin D deficient children with obesity but could be upregulated by vitamin D supplementation independently of the BMI. In vitro experiments in adipocytes conducted in this trial suggested that the mechanism of vitamin D control over adiponectin seems to occur post-transcriptionally [55]. Thus, the overall poor 25(OH)D status in our population and the lower adiponectin concentrations in children with overweight/ obesity compared to those with normal weight may have attenuated the association in children with overweight/ obesity in our study. On the other hand, we cannot rule out that insufficient power may have been responsible for null associations in this smaller group of children with overweight/ obesity. Similarly, the association between vitamin D levels and the inflammation score was weakened in the present analysis, potentially influenced by the chronic low-grade inflammatory status prevalent in children with excess body weight [25, 26].

Further, in children with overweight/ obesity, we observed a positive association between 25(OH)D with pro-inflammatory marker IP-10. IP-10 secretion is induced by leptin [56] and triggers the Th1 inflammatory process, while vitamin D can polarize this response towards the more regulatory Th2 response [57]. In vitro, vitamin D can suppress the Th1-related IP-10 in a dose-dependent manner in human monocytes [58]. Accordingly, correction of vitamin D insufficiency or deficiency after 6 weeks of supplementation decreased serum IP-10 concentrations, compared to placebo, in patients with chronic hepatitis [59]. Our unexpected finding of a positive association between 25(OH)D and IP-10 requires further investigation, although it may also be due to the high prevalence of insufficient and deficient vitamin D status in our population preventing a favourable effect on IP-10 concentrations.

We observed stronger associations between 25(OH)D and adiponectin in girls than in boys. Female sex has been shown to be independently associated with higher adiponectin concentrations in a cohort of Saudi children and adolescents [51]. In the same study, sex differences for the concentrations of several cytokines were reported, i.e. adiponectin, leptin and CRP concentrations seem to be higher, whereas TNF-α concentrations seem to be lower in girls than in boys [51]. Such sex differences may also have contributed to the observed positive association between 25(OH)D and TNF-α in girls but not in boys and the stronger negative association with the inflammation score in girls than in boys in our study. The latter indicates that girls may benefit more from achieving a normal vitamin D status based on its anti-inflammatory role.

### Limitations and strengths

An important strength of our study is its longitudinal design, providing associations between serum 25(OH)D and inflammatory markers within a large cohort of children and adolescents. This longitudinal design enables us to analyze these associations over two time points, thus offering stronger evidence as compared to cross-sectional studies. Unlike many experimental intervention studies that often focus on short-term vitamin D supplementation, our current study design enables us to assess long-term associations.

Our pan-European cohort, encompassing participants from Northern, Eastern, Western and Southern regions across Europe, provides high external validity. The participants were deeply phenotyped in terms of anthropometric measurements, biomarker data and health-related behaviours. Additionally, the reasonable sample size of the study allowed us to test non-linear associations. However, our analyses may have suffered from limited statistical power in stratified analyses, such as those assessing associations in children with overweight/obesity or when vitamin D levels were classified into different categories. Although we adjusted for the most relevant confounders, some influencing factors required the use of self-reported proxy markers. For instance, sports club membership served as a proxy for physical activity. Studies have shown that sports club members are more likely to reach the recommended time spent in moderate-to-vigorous physical activity (MVPA) [60] and that membership is associated with objectively measured MVPA [61] and vigorous physical activity [60]. Similarly, we used screen time per week as a marker for sedentary behaviour, which is associated with accelerometer-derived objectively measured sedentary time [62]. Further, there may have been information bias due to social desirability, particularly concerning sensitive information like smoking or alcohol consumption. However, research suggests that young adolescents report their alcohol consumption relatively reliably [63]. We used the month of blood draw and region of residence as markers of UV radiation exposure which can only roughly indicate the true exposure. Nevertheless, serum 25(OH)D concentrations may have already accounted for other UV-dependent factors such as dietary intake of vitamin D, time spent outdoors and skin pigmentation.

Finally, high quality standards were ensured by following standardized protocols and standard operating procedures for all measurements and examinations and by monitoring adherence of all centers by means of central trainings for field personnel and site visits.

## Conclusion

Our study demonstrated that higher vitamin D status is associated with favourable inflammatory profiles, particularly increased adiponectin and reduced inflammation score in children and adolescents. These associations are more pronounced in girls and in children with normal weight status. These findings suggest that improving vitamin D status may help regulate inflammatory markers. Given that almost all children in our cohort had an insufficient or deficient vitamin D status, public health measures should address widespread vitamin D deficiency to improve the inflammation profile in this population. However, the benefits of improved vitamin D status may be limited in children with excess body weight unless weight management is also addressed.

## Supplementary Information

Below is the link to the electronic supplementary material.

Supplementary file1 (DOCX 458 KB)

## Data Availability

Due to the sensitive nature of data collected from children and adolescents, ethical restrictions prohibit the authors from making the minimal data set publicly available. Each cohort center received approval of the corresponding local Ethical Commission. Data are available on request and all requests need approval by the study’s Steering Committee. Interested researchers can contact the study coordinator (ahrens@leibniz-bips.de or i.family@leibnizbips.de) to request data access. All requests for accessing data of the DEFIES/I.Family cohort are discussed on a case-by-case basis by the Steering Committee.
