# Supplementary material for: Longitudinal associations between vitamin D status and biomarkers of inflammation in a pan-European cohort of children and adolescents
Source: Eur J Nutr. 2024 Sep 4;63(8):3047–60. doi: 10.1007/s00394-024-03488-7 (PMC11519216; doi:10.1007/s00394-024-03488-7)
Supplement: Supplementary file 1 — Supplementary file1 (DOCX 458 KB) [file 394_2024_3488_MOESM1_ESM.docx]

**Supplementary Figures and Tables**

**Wolters et al.: Longitudinal associations between vitamin D status and biomarkers of inflammation in a pan-European cohort of children and adolescents**

T_3_: 01/2013 – 06/2014

(2^nd^ Follow-up examination)

T_1_: 09/2009 – 06/2010

(1^st^ follow-up examination)

T_0_: 09/2007 – 05/2008

(Baseline examination)

16 229 children (51% of those invited) valid for inclusion at T_0_

Time of examination Cohort Participation

11 041 (68% of T_0_)

participated at T_1_

6055 (55% of T_1_) participated at T_3_

+

2555 new recruitment

1050 participated at T_3_

2512 new recruitment

+

+

Study participants with 25-hydroxyvitamin D (25(OH)D) and at least 1 inflammatory marker available from T_0_/T_3_^a^ (n=1997)

- Intake of corticosteroids/anti-inflammatory drugs at cohort entry/follow-up examinations (n=188)
- Infections/other diseases indicated by CRP ≥ 10 mg/l at cohort entry/follow-up examinations (n=227)

Analysis dataset (N=1582)

at T_0_ & T_3_ = 633; T_0_ / T_3_ = 949

**Supplementary Fig 1**: Flowchart showing the study population and exclusion criteria

^a^25(OH)D and inflammatory markers were tested only at T_0_ and T_3_ and not at T_1_.


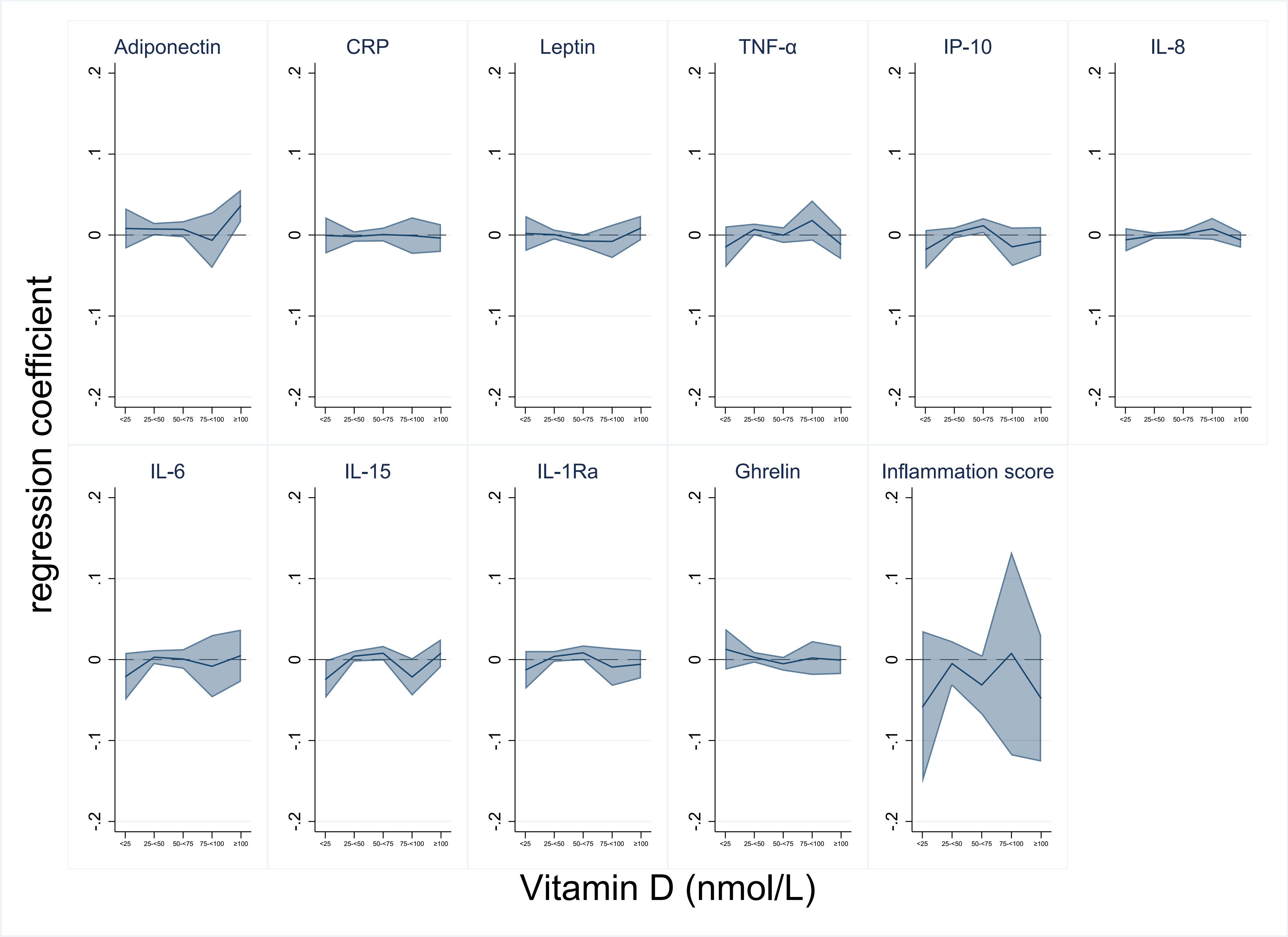


**Supplementary Fig 2**: Non-linear association between 25-hydroxyvitamin D levels and inflammatory markers using linear spline function with knots at 25, 50, 75 and 100 nmol/l. Abbreviations: 25(OH)D, 25-hydroxyvitamin D; CI, confidence interval; CRP, C-reactive protein; IL, interleukin; IL-1Ra, interleukin-1 receptor antagonist; IP-10, interferon gamma inducible protein; TNFα, tumor necrosis factor alpha. Inflammation score = sum of *z*‐scores of proinflammatory markers (CRP, leptin, TNF‐α, IP‐10, IL‐8, IL‐6) ‐ sum of *z*‐scores of anti‐inflammatory markers (IL‐1Ra, IL-15, adiponectin, ghrelin). Associations are adjusted for age, sex, study region, lifetime smoking and alcohol status, membership in sports club, screen time/week, BMI, parental education status and month of blood sample collection with age as a random slope.

**Supplementary Table 1**: Methods for the assessment of early life factors

| **Early life factor** | **Assessment methods** |
| --- | --- |
| Weight status of the mother | Maternal weight and height were self-reported and the BMI was calculated as weight (kg) divided by height (m) squared. |
| Preterm birth | Based on the gestational week of birth reported by the mothers, a binary variable was used: delivered at term vs. born preterm, i.e. ≤37^th^ week of gestation. |
| Birth weight (g) | Mothers reported the birth weight of the child. |
| Breastfeeding duration (months) | The total breastfeeding duration including combinations of breastfeeding with infant formula and food was reported by the mothers. |

**Supplementary Table 2**: Serum concentrations of inflammatory markers by weight status at baseline and follow-up examination

| **Inflammatory markers** | **Baseline examination (T_0_)** | | | **Follow-up examination (T_3_)** | | |
| --- | --- | --- | --- | --- | --- | --- |
|  | **Underweight** | **Normalweight** | **Overweight/ obese** | **Underweight** | **Normalweight** | **Overweight/ obese** |
| Adiponectin (µg/ml) | 25.10 (21.70; 34.13) | 24.65 (18.77; 33.51) | 21.68 (16.78; 30.49) | 24.31 (17.11; 41.18) | 22.85 (16.48; 32.58) | 18.63 (13.24; 30.08) |
| Leptin (ng/ml) | 1.04 (0.66; 1.69) | 1.43 (0.90; 2.42) | 4.71 (2.58;8,97) | 1.88 (1.13; 3.94) | 3.73 (1.85; 7.60) | 19.22 (8.10; 30.94) |
| Ghrelin (pg/ml) | 64.86 (26.03; 116.79) | 53.21 (24.60; 89.02) | 59.45 (32.57; 101.06) | 49.10 (24.49; 79.66) | 38.60 (16.81; 68.50) | 34.84 (16.27; 63.55) |
| CRP (mg/l) | 0.88 (0.26; 2.05) | 0.94 (0.32; 2.45) | 1.56 (0.58; 3.75) | 0.12 (0.04; 0.26) | 0.20 (0.07;  0.64) | 0.69 (0.29;  1.65) |
| IL-1Ra (pg/ml) | 324.98 (200.01; 462.62) | 308.13 (198.41; 446.87) | 361.64 (272.82; 517.86) | 222.52 (175.36; 276.45) | 253.91 (190.24; 332.27) | 358.64 (264.20; 524.04) |
| IL-6 (pg/ml) | 0.30 (0.16; 0.56) | 0.27 (0.17; 0.46) | 0.31 (0.19; 0.62) | 0.37 (0.26; 0.56) | 0.38 (0.26; 0.54) | 0.49 (0.35; 0.72) |
| IL-8 (pg/ml) | 3.32 (2.40; 4.87) | 3.21 (2.25; 4.55) | 2.93 (2.18; 4.38) | 6.65 (4.98; 9.27) | 6.34 (4.59; 9.18) | 6.25 (4.70; 8.82) |
| IL-15 (pg/ml) | 1.91 (1.35; 2.73) | 1.88 (1.27; 2.61) | 1.77 (1.09; 2.59) | 2.19 (1.77; 2.76) | 2.31 (1.80; 2.90) | 2.28 (1.72; 2.93) |
| IP-10 (ng/ml) | 0.18 (0.13; 0.25) | 0.18 (0.13; 0.26) | 0.17 (0.13; 0.28) | 0.20 (0.16; 0.27) | 0.21 (0.16; 0.30) | 0.22 (0.16; 0.30) |
| TNF-α (pg/ml) | 2.32 (1.74; 3.01) | 2.21 (1.67; 2.93) | 2.07 (1.78; 2.70) | 2.25 (1.91; 3.05) | 2.44 (1.99; 3.15) | 2.55 (1.99; 3.21) |
| Inflammation score, z-score | -0.47 (-1.02; 0.21) | -0.26 (-1.47; 0.80) | 1.11 (-0.30; 2.94) | -1.08 (-1.88; 0.29) | -0.46 (-1.49; 0.71) | 0.64 (-0.95; 2.19) |

Abbreviations: IL, interleukin; IL-1Ra, interleukin-1 receptor antagonist; IP-10, interferon gamma inducible protein; TNF-α, tumor necrosis factor alpha. Serum concentrations of

inflammatory markers are presented as median (25^th^ and 75^th^ percentiles).

| **Supplementary Table 3**: Association between 25-hydroxyvitamin D and inflammatory markers after additionally adjusting for early life markers and diet quality | | |
| --- | --- | --- |
| **Inflammatory markers** | **n** | ***β* (95% CI)** |
| Adiponectin | 1009 | **0.11 (0.07; 0.16)** |
| Leptin | 1258 | -0.02 (-0.05; 0.02) |
| Ghrelin | 976 | 0.00 (-0.04; 0.04) |
| CRP | 1256 | -0.01 (-0.05; 0.03) |
| IL-1Ra | 1227 | 0.03 (-0.01; 0.07) |
| IL-6 | 1214 | 0.00 (-0.05; 0.05) |
| IL-8 | 1228 | -0.01 (-0.03; 0.01) |
| IL-15 | 1228 | 0.02 (-0.02; 0.06) |
| IP-10 | 1228 | 0.01 (-0.03; 0.05) |
| TNF-α | 1228 | 0.03 (-0.02; 0.07) |
| Inflammation score | 711 | **-0.27 (-0.45; -0.08)** |
| Abbreviations: 25(OH)D, 25-hydroxyvitamin D; CI, confidence interval; CRP, C-reactive protein; IL, interleukin; IL-1Ra, interleukin-1 receptor antagonist; IP-10, interferon gamma inducible protein; TNFα, tumor necrosis factor alpha. Inflammation score = sum of *z*‐scores of proinflammatory markers (CRP, leptin, TNF‐α, IP‐10, IL‐8, IL‐6) ‐ sum of *z*‐scores of anti‐inflammatory markers (IL‐1Ra, IL‐15, adiponectin, ghrelin). The *ß* coefficient represents the *ß* unit change in the z-score of inflammatory markers per 12.5 nmol/l increase in 25(OH)D. Associations at *p* < 0.05 are shown in bold. Associations are adjusted for age, sex, study region, lifetime smoking and alcohol status, membership in sports club, screen time/week, BMI, month of blood sample collection, parental education status, pubertal status, healthy diet adherence score, birth weight, duration of breastfeeding, preterm birth, maternal obesity with age as a random slope. | | |

|  | | **Supplementary Table 4**: Association between 25-hydroxyvitamin D and inflammatory markers stratified by BMI   \| **Inflammatory markers** \| **Normal weight** \| \| **Overweight/obese** \| \| **Interaction with BMI** \| \| \| --- \| --- \| --- \| --- \| --- \| --- \| --- \| \| **n** \| ***β* (95% CI)** \| **n** \| ***β* (95% CI)** \| \| ***β* (95% CI)** \| \| Adiponectin \| 848 \| **0.12 (0.08; 0.17)** \| 236 \| 0.03 (-0.08; 0.15) \| \| **-0.11 (-0.22; -0.01)** \| \| Leptin \| 1076 \| 0.00 (-0.03; 0.03) \| 313 \| -0.12 (-0.26; 0.02) \| \| **-0.21 (-0.29; -0.14)** \| \| Ghrelin \| 862 \| -0.01 (-0.05; 0.04) \| 199 \| 0.02 (-0.04; 0.08) \| \| 0.07 (-0.02; 0.16) \| \| CRP \| 1071 \| 0.01 (-0.03; 0.05) \| 316 \| -0.09 (-0.22: 0.03) \| \| **-0.11 (-0.20; -0.03)** \| \| IL-1Ra \| 1046 \| 0.03 (-0.01; 0.08) \| 306 \| 0.01 (-0.10; 0.12) \| \| 0.00 (-0.09; 0.09) \| \| IL-6 \| 1031 \| 0.01 (-0.04; 0.07) \| 305 \| 0.07 (-0.13; 0.28) \| \| 0.00 (-0.13; 0.14) \| \| IL-8 \| 1048 \| 0.00 (-0.02; 0.02) \| 306 \| 0.01 (-0.02; 0.04) \| \| -0.01 (-0.07; 0.04) \| \| IL-15 \| 1047 \| 0.02 (-0.02; 0.06) \| 305 \| 0.01 (-0.08; 0.10) \| \| 0.03 (-0.06; 0.11) \| \| IP-10 \| 1048 \| 0.00 (-0.05; 0.05) \| 306 \| **0.09 (0.00; 0.18)** \| \| **0.11 (0.02; 0.20)** \| \| TNF-α \| 1047 \| 0.03 (-0.01; 0.08) \| 306 \| 0.08 (-0.01; 0.17) \| \| 0.00 (-0.10; 0.09) \| \| Inflammation score \| 612 \| **-0.21 (-0.39; -0.04)** \| 137 \| -0.17 (-0.53; 0.18) \| \| -0.19 (-0.57; 0.19) \| |  |
| --- | --- | --- | --- |
|  | Abbreviations: 25(OH)D, 25-hydroxyvitamin D; CI, confidence interval; CRP, C-reactive protein; IL, interleukin; IL-1Ra, interleukin-1 receptor antagonist; IP-10, interferon gamma inducible protein; TNFα, tumor necrosis factor alpha. Inflammation score = sum of *z*-scores of proinflammatory markers (CRP, leptin, TNF-α, IP‐10, IL‐8, IL‐6) ‐ sum of *z*‐scores of anti‐inflammatory markers (IL‐1Ra, IL‐15, adiponectin, ghrelin). Normal weight children: 18.5 ≤ BMI < 25; Overweight/obese children: BMI ≥25 (Cole & Lobstein 2012). The *ß* coefficient represents the *ß* unit change in the z-score of inflammatory markers per 12.5 nmol/l increase in 25(OH)D. Associations at *p* <0.05 are shown in bold. The model is adjusted for age, sex, study region, lifetime smoking and alcohol status, membership in sport club, screen time/week, month of blood sample collection and parental education status. p-value for interaction with BMI. | | |

**Supplementary Table 5**: Association between 25-hydroxyvitamin D and inflammatory markers stratified by sex

Abbreviations: 25(OH)D, 25-hydroxyvitamin D; CI, confidence interval; CRP, C-reactive protein; IL, interleukin; IL-1Ra, interleukin-1 receptor antagonist; IP-10, interferon gamma inducible protein; TNFα, tumor necrosis factor alpha. Inflammation score = sum of *z*-scores of proinflammatory markers (CRP, leptin, TNF-α, IP-10, IL-8, IL-6) - sum of *z*-scores of anti-inflammatory markers (IL-1Ra, IL-15, adiponectin, ghrelin). The *ß* coefficient represents the *ß* unit change in the z-score of inflammatory markers per 12.5 nmol/l increase in 25(OH)D. Associations at p <0.05 are shown in bold. The model is adjusted for age, study region, lifetime smoking and alcohol status, membership in sport club, screen time/week, BMI, month of blood sample collection and parental education status. *p*-value for interaction with sex

| **Inflammatory markers** | **Boys** | | **Girls** | | **Interaction with sex** |
| --- | --- | --- | --- | --- | --- |
|  | **n** | ***β* (95% CI)** | **n** | ***β* (95% CI)** | **β (95% CI)** |
| Adiponectin | 596 | **0.09 (0.03; 0.14)** | 554 | **0.16 (0.10; 0.23)** | 0.04 (-0.04; 0.12) |
| Leptin | 749 | -0.02 (-0.07; 0.03) | 681 | -0.03 (-0.08; 0.01) | -0.02 (-0.08; 0.03) |
| Ghrelin | 586 | 0.01 (-0.02; 0.03) | 533 | 0.03 (-0.01; 0.07) | 0.02 (-0.04; 0.09) |
| CRP | 749 | -0.01 (-0.06; 0.04) | 677 | -0.02 (-0.08; 0.03) | -0.01 (-0.07; 0.06) |
| IL-1Ra | 730 | 0.03 (-0.01; 0.08) | 663 | 0.02 (-0.04; 0.08) | 0.02 (-0.05; 0.08) |
| IL-6 | 722 | 0.00 (-0.04; 0.04) | 658 | 0.02 (-0.04; 0.09) | 0.05 (-0.04; 0.15) |
| IL-8 | 733 | 0.01 (-0.03; 0.04) | 663 | -0.01 (-0.02; 0.01) | -0.03 (-0.07; 0.01) |
| IL-15 | 733 | 0.01 (-0.04; 0.06) | 663 | 0.02 (-0.03; 0.08) | 0.03 (-0.04; 0.09) |
| IP-10 | 733 | 0.05 (0.00; 0.09) | 663 | -0.01 (-0.07; 0.05) | -0.04 (-0.11; 0.03) |
| TNF-α | 733 | 0.01 (-0.05; 0.07) | 663 | **0.06 (0.01; 0.12)** | 0.03 (-0.04; 0.10) |
| Inflammation score | 423 | -0.17 (-0.40; 0.05) | 387 | **-0.44 (-0.66; -0.22)** | **-0.30 (-0.59; -0.01)** |
